# Moving Together: Livestream Virtual Group Movement Classes for Persons With Dementia and Caregivers

**DOI:** 10.1093/geroni/igaa057.880

**Published:** 2020-12-16

**Authors:** Deborah Barnes, Cynthia Benjamin, Amanda Lee, Jennifer Lee, Wolf Mehling, Margaret Chesney, Rebecca Sudore

**Affiliations:** 1 San Francisco VA Health Care System, San Francisco, California, United States; 2 Together Senior Health, San Francisco, California, United States; 3 University of California,San Francisco, San Francisco, California, United States; 4 University of California, San Francisco, San Francisco, California, United States

## Abstract

MOVING TOGETHER classes deliver an online live-streaming version of the evidence-based Paired PLIÉ (Preventing Loss of Independence through Exercise) group movement program for people living with dementia (PLWD) and caregivers (CGs) that targets physical movements, mindful body awareness, and social engagement. Paired PLIÉ has been found to be associated with improvements in physical, cognitive, social/emotional function, and relationship quality. However, CGs also reported logistical challenges related to attending in-person classes. Our goal was to assess feasibility and satisfaction with an online version of the program developed by Together Senior Health, Inc. Fifteen of twenty-one participants (70%; 8 PLWD, 7 CGs) successfully completed the 12-week program, of whom all (100%) reported being highly satisfied with online delivery. Participants described physical, social and emotional benefits of participating: “I liked the activities with partner and that I could feel various parts of my body getting massage and movement (PLWD);” and “Really enjoyed this class, so did my body, brain and my spirit (CG).” Participants strongly supported the online format: “[I liked] BEING AT HOME! …Getting ready to go out, going out and coming home takes a lot of energy and we are not sure we would participate if it were not online (PLWD).” In conclusion, we found that online delivery of Moving Together is feasible and acceptable to PLWD and CGs. Offering livestream virtual group movement classes such as Moving Together could enhance accessibility to evidenced-based programs for PLWD and CGs.

